# The quality of feedback during formative OSCEs depends on the tutors’ profile

**DOI:** 10.1186/s12909-016-0815-x

**Published:** 2016-11-15

**Authors:** Noelle Junod Perron, Martine Louis-Simonet, Bernard Cerutti, Eva Pfarrwaller, Johanna Sommer, Mathieu Nendaz

**Affiliations:** 1 Unit of Development and Research in Medical Education, Faculty of Medicine, University of Geneva, Geneva, Switzerland; 2 Division of Primary Care Medicine, Department of Community Medicine, Primary Care and Emergencies, Geneva University Hospitals, 4 rue Gabrielle Perret-Gentil, 1211 Geneva 14, Switzerland; 3 Service of General Internal Medicine, Department of General Internal Medicine, Rehabilitation and Geriatric Medicine, Geneva University Hospitals, Geneva, Switzerland; 4 Unit of Primary Care Teaching and Research, Faculty of Medicine, University of Geneva, Geneva, Switzerland

**Keywords:** Formative OSCE, Quality, Feedback, Tutor

## Abstract

**Background:**

During their pre-clinical years, medical students are given the opportunity to practice clinical skills with simulated patients. During these formative objective structured clinical encounters (OSCEs), tutors from various backgrounds give feedback on students’ history taking, physical exam, and communication skills. The aim of the study was to evaluate whether the content and process of feedback varied according to the tutors’ profile.

**Methods:**

During 2013, all 2^nd^ and 3^rd^ year medical students and tutors involved in three formative OSCEs were asked to fill in questionnaires, and their feedback sessions were audiotaped. Tutors were divided into two groups: 1) generalists: primary care, general internist and educationalist physicians 2) specialists involved in the OSCE related to their field of expertise. Outcome measures included the students’ perceptions of feedback quality and utility and objective assessment of feedback quality.

**Results:**

Participants included 251 medical students and 38 tutors (22 generalists and 16 specialists). Students self-reported that feedback was useful to improve history taking, physical exam and communication skills. Objective assessment showed that feedback content essentially focused on history taking and physical exam skills, and that elaboration on clinical reasoning or communication/professionalism issues was uncommon. Multivariate analyses showed that generalist tutors used more learner-centered feedback skills than specialist tutors (stimulating student’s self-assessment (*p* < .001; making the student active in finding solutions, *p* < .001; checking student’s understanding, *p* < .001) and elaborated more on communication and professionalism issues (*p* < 0.001). Specialists reported less training in how to provide feedback than generalists.

**Conclusion:**

These findings suggest that generalist tutors are more learner-centered and pay more attention to communication and professionalism during feedback than specialist tutors. Such differences may be explained by differences in feedback training but also by differences in practice styles and frames of references that should be further explored.

**Electronic supplementary material:**

The online version of this article (doi:10.1186/s12909-016-0815-x) contains supplementary material, which is available to authorized users.

## Background

The objective structured clinical examination (OSCE) is designed to evaluate learners’ skills such as history taking, physical examination, communication and professionalism. Objective assessment is important because self-assessment alone is often inaccurate and insufficient for the development of clinical expertise [1–3].

OSCEs may be used to evaluate performance for both formative and summative purposes. Formative feedback is defined as “information communicated to the learner that is intended to modify his or her thinking or behavior for the purpose of improving learning” [4]. Formative feedback reinforces appropriate learning and contributes to correction of deficiencies and to learners’ self-monitoring [5]. It is especially effective when information about previous performance is used to promote positive and desirable development [6]. Studies have shown that immediate feedback to students after OSCEs leads to quick and sustained improvement in subsequent performance [7] and enhances students’ self-assessment skills [8].

Feedback effectiveness is influenced by a number of factors, including tutor-student interactions, the feedback message, and the environment in which feedback takes place [3, 6]. Feedback seems to be more effective when learners are more oriented towards learning than performance demonstration [4, 6, 9, 10], and when the feedback content matches some of the learners’ self-perceptions [11]. Feedback is also more effective when it is based on observed facts, focuses on tasks, is specific, concise, and suggests areas of improvement [12]. Feedback may be directive—the tutor tells the learner what to do or improve – or it may be facilitative and elaborative - the tutor makes suggestions to guide learners in self-reflection about the encounter and their skills [3, 4]. Feedback also seems to be better accepted when the tutor is perceived as being credible [3, 6, 13] and is comfortable with the topic [14]. However, the relationship between students’ learning styles and their use of feedback in the specific context of an OSCE requires further study [15].

The personal and professional characteristics of tutors who give feedback to students during formative OSCEs may influence the way feedback is delivered and perceived. In a British study that looked at the quality of feedback on mini CEX (a 10–20 min structured assessment of an observed clinical encounter), tutors who were academic trainees (clinical or research fellows) focused more on positive aspects of performance, provided more suggestions and more often recorded an action plan than consultants or clinical trainees such as specialist registrars and senior house officers [16]. A study assessing the quality of formative feedback provided by different types of medical examiners showed that senior students received higher feedback ratings than faculty [8]. Surveys among Geneva University medical students suggest a preference for OSCE stations involving feedback from generalists as compared with specialists (personal communication). However, the influence of tutors’ characteristics on the content and delivery of feedback has been studied mainly through subjective reports, and is still debated.

The aims of our study were: 1) to evaluate students’ perceptions of the feedback they received after a formative OSCE; 2) to objectively assess the quality of the feedback; and 3) to explore the differences between feedback provided by “specialists” (content experts) and “generalists”. We were especially interested in evaluating to what extent the content and the process of feedback varied according to the tutors’ profiles.

## Methods

### Design, setting and participants

We conducted a prospective study at the Faculty of Medicine of Geneva University, Switzerland. The Faculty of medicine offers a 6-year curriculum to about 150 students per year. The curriculum is divided into three pre-clinical years (bachelor) and three clinical years (master). During the 2^nd^ and 3^rd^ pre-clinical years, medical students are required to practice clinical skills such as history taking, physical examination and communication skills during four formative OSCEs which have two different formats:

1. A 20- min interaction with a simulated patient observed by a tutor, followed by a 15-min feedback session (direct observation). The observation takes place behind a two-way mirror.
2. A 20-min videotaped interaction with a simulated patient, followed by a delayed 40-min feedback session by the tutor (including observation of the videotaped consultation), after the student has reviewed and analyzed his own performance (video based).

All students go through a minimum of two “direct observation” and one “video-based” sessions over two years. Attribution is made automatically and does not depend on students’ preferences. The first OSCE session provides only “direct observation” feedback.

Generally, 20 to 30 physicians are involved as tutors for direct observation and video based feedback. Tutors include experienced clinicians who have both clinical and teaching responsibilities and are consultants, registrars or senior house officers whatever their discipline. About one-third are specialists and two-thirds are generalists (general internists working in outpatient or inpatient care), or educationalists working as part- or full-time program coordinators in the medical school. Most tutors regularly interact with students during formal course activities given in 2^nd^ and 3^rd^ year. Specialists are involved in the OSCE related to their field of expertise and mainly give feedback after direct observation (only a few occasionally provide video-based feedback). Generalists may be involved in all OSCEs and half of them give both direct and video-based feedback.

Although there is no required faculty training for these formative OSCEs, all tutors are requested to attend two mandatory basic workshops on their role as tutor for small group and one-to-one teaching activities during medical school. In addition, all tutors have the option to attend a general training session on feedback provided every year by the medical school as part of a faculty development program. Before each OSCE, all tutors receive written information about the goals of the OSCE, the elements of effective feedback and what is expected from them as tutors.

During observation, the tutors fill in a checklist (including items on history taking, physical examination, quality of explanation given to the patient, the way the encounter is ended) which is not a summative assessment, but rather is used to provide feedback. At the end of the feedback session, the tutor documents on a paper portfolio the strengths and weaknesses of the student’s performance and makes suggestions for the next formative OSCE in order to inform the next tutor and help define the student’s needs. Beyond this paper portfolio, no other information is given to students about their performance from one OSCE to the other.

### Data collection

During the 2012–2013 academic year, all 2^nd^ and 3^rd^ year medical students who undertook “direct observation” and “video-based” formative OSCEs were invited to participate in the study. All physicians involved as tutors were also invited to participate. OSCE stations focused on gastrointestinal, cardiac, and neurological topics. All feedback sessions were audiotaped. Students’ age and gender were recorded, as well as tutors’ socio-demographic characteristics and clinical and teaching experiences (Table 1).

**Table 1:** Tutors’ sociodemographic data, clinical and teaching experience

Socio-demographic data and clinical and teaching experience	All	Generalists	Specialists	p
	n=38	n=22	n=16	
Female n (%)	17 (45%)	13 (59%)	4 (25%)	0.079
Mean age (SD)	45 (8)	44 (9)	48 (5)	0.123
Mean years of clinical experience (SD)	18 (8)	17 (10)	20 (6)	0.341
Mean years of clinical supervision (SD)	9 (7)	8 (7)	11 (6)	0.133
Mean years as OSCE tutor (SD)	4 (4)	5 (4)	4 (3)	0.642
Involved in communication skills teaching n (%)	16 (42%)	14 (64%)	2 (12%)	0.005
Structured training in feedback skills n (%)	28 (74%)	19 (86%)	9 (56%)	0.062

The overall project was approved by the research ethics committee of the University Hospitals of Geneva. A complete review was waived by this committee. However, all participants signed up a written consent form.

### Outcome measures

1. *Students’ perceived utility and quality of the feedback* was measured with a 15 item questionnaire (1–5 Likert scale), administered immediately after the feedback session. The items focused on the usefulness of the feedback for improving skills in history taking, physical examination and communication, as well as on the different elements of the feedback process (self-evaluation, active learning, checking understanding, etc.…) (Table 2). We tested the questionnaire items in a pilot survey of 26 students after a formative OSCE (respiratory topic), and confirmed their ability to discriminate between good and poor feedback providers.

**Table 2:** Quality of the feedback perceived by the students (*n* = 348 questionnaires, 79 “video based” and 269 “direct observation” feedback sessions)

Quality of the feedback perceived by the students	All Tutors	Generalists	Specialists		
	*n* = 38	*n* = 22	*n* = 16		
(Likert 1: completely disagree-5: completely agree)	Mean (SD)	Mean (SD)	Mean (SD)	p	Adjusted p*
The feedback session was useful	4.73 (0.57)	4.78 (0.49)	4.63 (0.60)	0.034	0.164
I improved my history taking skills	4.35 (0.84)	4.38 (0.79)	4.26 (0.88)	0.250	0.435
I improved my physical examination skills	4.26 (0.94)	4.30 (0.92)	4.12 (1.00)	0.162	0.306
I improved my communication skills	4.20 (0.93)	4.26 (0.87)	3.97 (1.10)	0.102	0.051
The tutor was aware of what I needed to learn	4.74 (0.58)	4.80 (0.48)	4.59 (0.72)	0.003	0.010
The tutor made me feel comfortable and confident	4.65 (0.72)	4.72 (0.61)	4.48 (0.83)	0.006	0.024
The tutor asked me my learning needs	3.56 (1.63)	3.81 (1.54)	2.75 (1.62)	<0.001	0.013
The tutor asked me to evaluate what I did well	4.51 (0.90)	4.59 (0.77)	4.30 (1.10)	0.014	0.096
The tutor asked me to evaluate what I could improve	4.74 (0.62)	4.83 (0.47)	4.56 (0.82)	0.001	0.005
The tutor gave me balanced feedback (including both positive and less positive aspects)	4.71 (0.64)	4.80 (0.47)	4.57 (0.85)	0.006	0.030
The tutor stimulated me to participate to the problem solving process	3.95 (1.24)	4.14 (1.13)	3.43 (1.36)	<0.001	0.001
The tutor gave me precise and concrete suggestions for improvement	4.47 (0.92)	4.53 (0.84)	4.30 (1.08)	0.065	0.128
The tutor provided me opportunities to practice parts of the history taking, physical exam or the communication	2.73 (1.62)	2.84 (1.60)	2.32 (1.63)	0.015	0.184
The tutor asked the simulated patient to give me a feedback	4.42 (1.29)	4.58 (1.11)	4.04 (1.57)	0.002	0.159
The tutor checked my understanding	3.23 (1.61)	3.49 (1592)	2.52 (1.48)	<0.001	0.001

*Using a model taking into account the type of OSCE (fixed effect), and the supervisor (random effect)
2. *Objective assessment of feedback quality* using a 21-item feedback scale developed for a previous study on feedback and adapted to the context of OSCEs (Likert scale 0–5) [17]. The feedback scale focused both on the content and process of the feedback. Content items (*n* = 7) were coded as described in Table 3 and were based on the checklist sections for the first four elements. Clinical reasoning and communication/professionalism issues were added by the investigators after having listened to several feedback sessions and identified that some tutors were addressing these issues. Items were counted but not explored qualitatively. Process, evaluated with a Likert scale (0–5), was coded according to the items displayed in Table 4 (*n* = 14): 9 describing specific elements of the feedback process, four transversal dimensions and a global rating. The scale structure was inspired by the MAAS-Global Score, a well-known communication skill coding instrument [18].

**Table 3:** Categories used to code the content of the feedback and related definitions

Category	Definition	Example
Content – history taking	Number of items mentioned and/or discussed regarding history taking during the feedback session	It is good that you asked about the presence of shortness of breath
		Don’t forget to ask about the past history
Content – physical examination	Number of items related to diagnosis and management mentioned and/or discussed regarding the physical examination during the feedback session	Your physical exam was systematic
		You did not palpate the spleen
Content – explanation and planning-ending the session	Number of items mentioned and/or discussed regarding the suspected diagnosis and management	The diagnosis was correct
Content – communication skills	Number of items related to communication skills mentioned and/or discussed during the feedback session	I liked the way you responded to the patients’ emotions
		You used jargon during the encounter
Elaboration – clinical reasoning	Number of times the tutor elaborated in directive or facilitative way on the importance or relevance of collecting such items during the feedback session	Why is it important to ask about gynecological complaints in a young woman with abdominal pain? (facilitative)
Elaboration – communication/professionalism	Number of times the tutor elaborated in directive or facilitative way on the importance of professionalism or communication	Do not forget to explore patients’ beliefs and emotions: it will influence the way you will explain the diagnosis (directive)

**Table 4:** Objective analysis of the quality of the feedback (*n* = 140 videotaped “direct observation” feedback sessions)

Objective analysis	All Tutors	Generalists	Specialists		
	*n* = 35	*n* = 21	*n* = 14		
	Mean (SD)	Mean (SD)	Mean (SD)	p	Adjusted p*
Content *(number of comments per feedback on...)*					
Global performance	0.64 (0.66)	0.54 (0.60)	0.77 (0.72)	0.039	0.264
History taking	4.76 (3.51)	5.73 (3.75)	3.54 (2.77)	<0.001	0.160
Physical examination	5.17 (3.05)	5.73 (3.17)	4.47 (2.76)	0.014	0.372
Explanation-end	1.01 (1.03)	0.92 (1.10)	1.13 (0.93)	0.242	0.169
Communication	2.30 (1.70)	2.61 (1.89)	1.92 (1.32)	0.015	0.230
Elaboration- clinical reasoning	1.56 (1.53)	1.76 (1.57)	1.32 (1.45)	0.09	0.768
Elaboration- communication/professionalism	1.46 (1.39)	1.96 (1.41)	0.82 (1.06)	<0.001	<0.001
Process *(Likert 0: completely disagree - 5: completely agree)*					
The tutor explored students’ learning needs	2.67 (1.53)	3.49 (0.90)	1.67 (1.54)	<0.001	<0.001
The tutor stimulated students’ self-assessment	2.30 (1.53)	3.07 (1.11)	1.37 (1.45)	<0.001	<0.001
The feedback was descriptive	3.63 (1.22)	4.12 (0.92)	3.00 (1.27)	<0.001	0.001
The feedback was subjective (using “I”)	3.16 (1.85)	3.99 (1.52)	2.11 (1.70)	<0.001	<0.001
The feedback was balanced	3.78 (1.27)	4.23 (0.90)	3.21 (1.44)	<0.001	<0.001
The supervisor took into account the student’s self-assessment	2.30 (1.65)	3.19 (1.27)	1.23 (1.42)	<0.001	<0.001
The tutor stimulated students to participate to the problem solving process	2.96 (1.13)	3.55 (0.86)	2.21 (0.98)	<0.001	<0.001
The tutor used role-playing or hands on to give students the opportunity to practice parts of the consultation	1.18 (1.30)	1.49 (1.38)	0.78 (1.08)	0.001	0.036
The tutor checked students’ understanding at the end of the idem	2.70 (1.60)	3.67 (1.14)	1.47 (1.20)	<0.001	<0.001
Transversal dimensions					
Empathy	3.92 (1.02)	4.46 (0.62)	3.24 (1.02)	<0.001	<0.001
Pedagogical effectiveness	3.14 (1.23)	3.86 (0.86)	2.23 (1.00)	<0.001	<0.001
Structure of the feed-back	3.11 (1.25)	3.95 (0.79)	2.05 (0.86)	<0.001	<0.001
Verbal interaction	3.33 (1.05)	3.74 (0.99)	2.81 (0.88)	<0.001	0.002
Global evaluation	3.30 (1.07)	4.01 (0.61)	2.40 (0.82)	<0.001	<0.001

*using a model taking into account the type of OSCE (fixed effect), and the supervisor (random effect)

The coding definitions were refined after reviewing 10 audiotaped feedback sessions (NJP, MLS, EP, MN). Then, NJP and MLS first double-coded 15 audiotaped feedback sessions to ensure appropriate understanding of the coding definitions and then independently coded the remaining audiotaped feedback sessions. Interrater reliability, calculated on the basis of 10% of the remaining audiotaped feedback sessions, was good (intraclass correlation coefficient = 0.82).

### Analysis

Sociodemographic data were described using percentages, or means and standard deviations (SD). Feedback content data were described as the mean number of items addressed per feedback session. Feedback process data were summarized by means (Likert scale) and standard deviations. Comparisons of categorical variables were made using Chi-square test or Fisher exact tests, and student’s *t*-test for continuous variables (such as feedback content and process). For the analysis of perceived feedback quality, adjusted *p*-values were computed using a model that was taking into account the type of OSCE (fixed effect), and the supervisor (random effect). For the analysis of the objective quality of feedback, only direct observation feedback sessions were included, since specialists were rarely involved in the video-based format. In complementary analyses, the association with other factors such as students’ gender, type of feedback (based on video or direct observation), tutors’ gender, tutors’ feedback training, tutors’ clinical experience, tutors’ involvement in communication skills teaching, and tutors’ OSCE experience, were also systematically investigated, using stepwise multivariate analysis.

All analyses were run on R 2.15.3 (the R Foundation for Statistical Computing), and TIBCO Spotfire S + ® 8.1 for Windows (TIBCO Software Inc).

## Results

### Sociodemographic and training data

All 251 medical students participated in the study (137 women, 55%), 163 from the 2^nd^ year and 88 from the 3^rd^ year of medical school. The smaller number of 3^rd^ year medical students is explained by the fact that the study included only 3 OSCEs out of 4 and that 3^rd^ year students only filled in the questionnaire for one feedback session . Out of 41, 38 tutors participated (22 generalists and 16 specialists): 3 specialist tutors refused. Specialist tutors were less involved in communication skills teaching, and tended to be more often male and less trained in feedback skills (Table 1).

### Medical students’ evaluation of the feedback

Medical students filled in 348 questionnaires after “video based” or “direct observation” feedback, some of them having attended two different formative OSCEs. Their perception of the feedback was globally very good (Table 2). All items were scored above four except for exploration of students’ needs, active involvement in problem solving, opportunities to practice, and checking understanding at the end of the feedback. There were no statistically significant differences between generalists’ and specialists’ feedback in the way students perceived its usefulness and impact on skill improvement. However, they reported that generalists involved them more actively than specialists at all stages of the feedback.

The overall quality of the feedback was lower for OSCEs in cardiology or neurology (differences of 0.26; *p* = 0.002) or when students had received a feedback after direct observation instead of based on video (difference of 0.39; *p* < 0.001).

#### Objective analysis of the feedback

Both content and delivery process of tutors’ feedback were objectively analyzed for 140 audiotaped “direct observation” feedback sessions. We found that feedback content focused on history taking and physical exam skills and to a lesser extent on communication skills and rarely included global comments on performance (Table 4). Elaboration on clinical reasoning or communication/professionalism issues was rare. Students’ active involvement in feedback, evaluated through items such as students’ exploration of learning needs, self-assessment, active participation in problem-solving, opportunities for practice and checking for understanding, was average to low. The mean duration of direct-observation based feedback was longer for generalists than for specialists (12.49 min versus 6.48; *p* = <0.001). Specialists scored lower in almost all items of the feedback process and addressed communication or professionalism issues less often.

Complementary multivariate analyses showed that, taking into account the type of OSCE, and apart from being a specialist, there were two additional factors that often negatively influenced the feedback process (i.e., defining learning needs, stimulating self-assessment, involving the student in the problem-solving checking understanding): lack of training in how to give feedback and shorter experience with OSCE supervision. Tutors with more clinical experience made more elaborate comments on clinical reasoning (*p* < 0.001).

## Discussion

In this study, we found that medical students highly valued formative OSCEs and perceived that the feedback helped them improve their skills in history taking, physical examination and communication, even when feedback was objectively assessed to be average to poor. Students may appreciate having experienced clinicians spending time with them and sharing their perspectives. However, the objective analysis of the quality of feedback showed that while tutors gave rather specific and descriptive feedback, they did not often actively involved students in the learning process. Tutors addressed biomedical issues, such as history taking and physical examination items, more often than clinical reasoning, communication and professionalism issues. Feedback from specialists and generalists differed in terms of content and process, even after adjusting for tutors’ age, gender, and clinical and teaching training and experience. Specialists (cardiologists, neurologists, gastroenterologists, visceral surgeons and anesthesiologists) addressed fewer elements in a shorter period of time and in a more directive way. Generalists elaborated more often on communication and professionalism issues and involved students more actively in the feedback process.

Tutors may benefit from training in how to more actively involve students during feedback. It has been shown that faculty members who question residents throughout the feedback process are able to learn more about the resident’s knowledge and skills than through observations alone [3]. Several faculty programs have been effective in improving teaching skills and encouraging a more learner-centered approach [19].

Tutors rarely elaborated on clinical reasoning and communication/professionalism issues during feedback on history taking, physical examination and communication. It has been said that “a good preceptor is one who challenges the student when giving feedback” [14] and one could have expected experienced clinicians to more actively stimulate students’ thinking and reasoning. While student raters may represent a potentially cheaper and more available alternative to physicians in feedback sessions [8, 20–22], we feel that involving clinically experienced physicians in formative OSCEs is important for several reasons: they have credibility as clinical experts; their clinical expertise and experience allow them to stimulate students’ clinical reasoning more effectively than student raters, and feedback experience during OSCEs may prepare clinical supervisors to give better and more regular feedback to residents in their regular work environment. However, in order to deliver effective feedback, tutors should have a variety of frameworks in mind to help students gain a deeper understanding of factors influencing their own reasoning or behaviors, revisit their assumptions and values and develop a broader range of possible responses and interventions [14, 23]. Faculty programs on feedback should not only focus on teaching skills but also on the acquisition of such conceptual frameworks in order to respond to students’ educational needs. Similarly, OSCEs may be an opportunity for students to not only practice history taking, physical examination and communication skills but also to link these skills to the clinical reasoning process.

In our study, objective assessment of feedback from specialists and generalists showed differences in terms of content and process. Similar differences in the feedback process were also reported by students, but to a lesser extent. Differences in teaching skills among different profiles of physicians or in different clinical settings have already been described. Ramsey et al. reported that teachers in the ambulatory setting received higher ratings of teaching effectiveness than those working in inpatient settings [24]. Studies from the United States found that students and resident rated hospitalists and general medicine attending physicians as more effective teachers than sub-specialist attending physicians on the inpatient wards [25, 26]. However, characteristics such as perceived involvement, enthusiasm, frequency of feedback delivery and familiarity with teaching principles appear to play a greater role than the affiliation, age or academic rank of the physician [16, 27, 28].

Observed differences between specialists and generalists should be interpreted with caution since there were a number of potential confounding variables (most specialists were males and not involved in feedback training). However, such differences may be explained by the fact that specialists were less trained to give feedback and may be consequently less aware that the role of a clinical teacher is to facilitate rather than to direct learning. It is also possible that the generalists, who included a high proportion of primary care physicians, may be more familiar with an interactive rather directive style of communication, given its importance in the patient-centered approach and its relevance in caring for ambulatory patients suffering from chronic illnesses. Indeed, learner-centered and patient-centered approaches are very similar [29] and one may hypothesize that skills used in one approach may translate into the other. Differences in feedback content between specialists and generalists may also result from the fact that tutors use different frameworks and weigh checklist items differently according to their specialty, practice style and level of comfort when evaluating students’ performance [30, 31]. From such a perspective, it is not surprising that specialists selected a higher proportion of cognitive issues and a lower proportion of communication issues from the checklist than generalists. Communication and professionalism require a frame of references that is often ignored by tutors by lack of training in this field and can be felt as more challenging than history taking or physical examination [30, 32]. However, it does not mean that specialist cannot achieve a more relationship-centered approach.

Our study has several limitations. First, students’ perception of feedback quality was assessed through a questionnaire. It would have been of interest to explore more qualitatively the way the content and process of the feedback influenced their appreciation. Second, the objective and quantitative analysis of the feedback content, which focused on counting the elements addressed during feedback, gives only a limited view of what was discussed during the session. Third, several confounding factors could have had an impact on the results, since the design was unbalanced: the first OSCE did not include any video format; and the proportion of generalists and specialists completing both types of feedback formats differed. However, we made a sub-analysis restricted to the generalist and specialist tutors who gave feedback in both formats: the conclusions were similar though the evidence was weaker. Finally, although intercoder reliability was high, raters were not completely blinded since they knew some of the tutors and could have recognized their voice while listening to the audio-taped feedback sessions.

## Conclusion

All tutors should be trained to systematically challenge students’ clinical reasoning and to address communication and professionalism during formative OSCEs. Differences in feedback quality between generalists and specialists may be explained not only by differences of training in teaching skills but also by use of different patterns of practice and frameworks. Future research should explore in more depth to which extent tutors’ clinical expertise, their type of practice and work setting, their interest in teaching and their perceptions of their role as OSCEs tutor influence their way of delivering feedback to students during formative OSCEs.

## Additional files

Additional file 1: Codes and definition of codes. (XLSX 15 kb)
Additional file 2: Contains the data about the quality of the feedback perceived by the students. (XLSX 41 kb)
Additional file 3: Contains the data about the tutors’ sociodemographic data, clinical and teaching experience. (XLSX 9 kb)
Additional file 4: Contains the data about the objective analysis of the quality of the feedback. (XLSX 33 kb)

## Abbreviations

EP: Eva Pfarrwaller
MLS: Martine Louis Simonet
MN: Mathieu Nendaz
NJP: Noelle Junod Perron
OSCE: Objective structured clinical examination
SD: Standard deviation
